# Activation of senescence in critically ill patients: mechanisms, consequences and therapeutic opportunities

**DOI:** 10.1186/s13613-023-01236-4

**Published:** 2024-01-05

**Authors:** Paula Martín-Vicente, Cecilia López-Martínez, Beatriz Rioseras, Guillermo M. Albaiceta

**Affiliations:** 1 Instituto de Investigación Sanitaria del Principado de Asturias, Oviedo, Spain; 2 Centro de Investigación Biomédica en Red (CIBER)-Enfermedades Respiratorias, Madrid, Spain; 3 Instituto Universitario de Oncología del Principado de Asturias, Universidad de Oviedo, Oviedo, Spain; 4 Servicio de Inmunología, Hospital Universitario Central de Asturias, Oviedo, Spain; 5 Unidad de Cuidados Intensivos Cardiológicos, Hospital Universitario Central de Asturias, Avenida del Hospital Universitario s/n, 33011 Oviedo, Spain

**Keywords:** Senescence, Senotherapeutics, Apoptosis, DNA damage response, Post-ICU syndrome

## Abstract

Whereas aging is a whole-organism process, senescence is a cell mechanism that can be triggered by several stimuli. There is increasing evidence that critical conditions activate cell senescence programs irrespective of patient’s age. In this review, we briefly describe the basic senescence pathways and the consequences of their activation in critically ill patients. The available evidence suggests a paradigm in which activation of senescence can be beneficial in the short term by rendering cells resistant to apoptosis, but also detrimental in a late phase by inducing a pro-inflammatory and pro-fibrotic state. Senescence can be a therapeutic target. The use of drugs that eliminate senescent cells (senolytics) or the senescence-associated phenotype (senomorphics) will require monitoring of these cell responses and identification of therapeutic windows to improve the outcome of critically ill patients.

## Introduction

Critical illness is often characterized by a systemic response beyond the primary site of injury. Severe insults trigger a large variety of responses, evolutionarily optimized and aimed to survive, that regulate essential cell processes such as active cell death (programmed or not), cell division, inflammation or regulation of metabolism [1]. The development of supportive techniques that preserve life despite massive injuries has improved early survival rates, but the persistent activation of those adaptative mechanisms turns them into pathogenetic, contributing to late organ dysfunction and death [2]. This dual nature of the response to aggression (tuned by evolution but also pathogenetic) difficults the identification of therapeutic targets that further improve the outcomes of our severe patients.

Senescence is defined as a cell state characterized by a stable arrest of cell cycle and a phenotypic change that includes the release of paracrine factors that, among other actions, promote inflammation and fibrosis [3]. Although senescence has often been linked to aging, it must be noted that any challenge to cell integrity, including DNA damage or breakdown, results in its activation irrespective of the age of the subject. Senescence is a paradigmatic example of a pro-survival mechanism, deeply rooted in our biochemical machinery, that may also be pathogenetic. In this review, we will discuss how a critical disease can promote accelerated aging by activating senescence mechanisms.

## An overview of senescence mechanisms

The term senescence was first used to refer to the finite proliferative capacity of primary fibroblasts observed in vitro [4]. This cell cycle arrest is now known to be a subtype of cellular senescence, termed replicative senescence, and caused by telomere shortening.

Nowadays, hundreds of different stimuli have been found to trigger a senescent response, and most of them are directly or indirectly related to the development of DNA damage and activation of the DNA damage response (DDR). These include ionizing radiation that cause double strand breaks [3], telomere shortening as consequence of multiple replication cycles [4], mitochondrial dysfunction and the consequent production of reactive oxygen species (ROS) [5], or activation of oncoproteins that lead to aberrant replication patterns [6]. Each one of these stimuli, either by direct activation of the DDR or by action of other cellular stress responses, lead to the activation of cyclin-dependent kinase inhibitors [6]. P53 is a master regulator of diverse cellular processes that becomes activated in response to these damages, and plays a part in deciding the fate of the cell towards apoptosis or senescence [7]. Following the senescence path, P53 will activate the cyclin-dependent kinase inhibitor p21, but other inhibitors such as p16, p27 or p15 can also participate in the cell cycle arrest. Due to this inhibition activity, the RB protein will remain dephosphorylated, allowing its action as inhibitor of the E2F transcription factor family and thus effectively arresting the cell cycle [8].

However, cell cycle arrest is not the only characteristic of senescent cells. The senescent phenotype involves other changes such as secretion of several mediators, increased lysosomal content, nuclear reorganization, apoptosis resistance, endoplasmic reticulum stress, metabolic reprogramming, changes in membrane components, or changes in cell size [9]. It’s important to highlight that not all these characteristics are found in every senescent cell, nor these processes are exclusive to senescent cells, which makes identifying and studying this cellular state a real challenge.

The development of a senescence-associated secretory phenotype (SASP) is one of the most studied characteristics of senescent cells because of its consequences [10]. Senescent cells secrete cytokines, chemokines and proteases that in a physiological state are aimed to promote tissue remodeling and attract immune cells that will clear the tissue of unwanted cells, including the senescent ones. However, in a pathological context in which there is no clearance of the senescent cells, the SASP will produce chronic inflammation and fibroblast activation leading to development of fibrosis [3]. This secretory phenotype is orchestrated by the transcription factors NF-κB and CEBPβ, and includes the release of IL-6, IL-8, monocyte chemoattractants, macrophage inflammatory proteins and growth factors such as TGFβ [11].

Another key characteristic of senescent cells is an increase in lysosomal content and proteins, including the lysosomal enzyme β-galactosidase. Increases in the quantity of this enzyme have been used as a surrogate marker for this change in lysosomal activity, and the positive assay of this enzyme at the suboptimal pH of 6.0 is considered a senescence marker, termed senescence-associated β-galactosidase [12, 13].

Changes in chromatin organization can also be observed in senescent phenotypes. Phosphorylation of histone H2AX occurs in sites of DNA breaks and drives the recruitment of DNA repair complexes. Also, heterochromatin foci form as a mechanism to repress proliferation-promoting genes, for which HP1γ is a marker [14, 15].

Of note, no specific marker of senescence has been validated in patients and using routinely available samples. This lack of biomarkers directly implies that identification and quantification of the “senescent state” of a tissue or individual in the clinical practice is an almost impossible task, and will have its consequences to identify therapeutic windows to manipulate senescence.

## Activation of senescence in critical illness

Critically ill patients face a series of challenges that arise from both the underlying disease and the necessary medical interventions for their treatment. This interaction between pathology and therapy activates a set of damage mechanisms that can lead to the development of complex pathophysiological responses, including cellular senescence.

There are different critical care scenarios where an increase of the senescent response has been observed. A widely studied trigger is ischemia/reperfusion injury [16], which refers to the damage caused by a temporary lack of blood supply followed by its restoration. This critical situation commonly occurs in emergency surgery, organ transplantation, and cardiovascular events. Hyperoxia per se can also activate senescence mechanisms [17–20]. In both situations, the main underlying mechanism that leads to cellular senescence is oxidative stress. In the case of ischemia, cells face a decrease in the supply of oxygen and essential nutrients. This oxygen deprivation can trigger the production of ROS. When blood is restored during reperfusion, cells may experience a sudden increase in the supply of oxygen and nutrients, that paradoxically, can lead again to an increase in ROS due to the release of stored free radicals during ischemia [21–23]. In the case of hyperoxia, prolonged exposure to elevated oxygen levels can increase ROS [24]. The production of reactive oxygen and nitrogen species can directly damage nucleic acids, thereby triggering the DDR and the following senescent response [25]. This makes oxidative stress a fundamental mechanism in the critically ill patient to address senescence not only in these two contexts but also across a wide range of scenarios.

Inflammation is another relevant mechanism. Systemic inflammation is not only a common feature of critical illnesses, such as viral infections [26], but can also arise as a consequence of their treatment, including interventions such as mechanical ventilation [27]. Inflammation initiates or intensifies cellular senescence through various mediators, such as IL-6. Additionally, senescent cells can contribute to systemic inflammation by their SASP, which includes proinflammatory cytokines, growth factors and proteolytic enzymes. Consequently, a positive feedback loop is established between inflammation and senescence [28, 29].

Despite its supportive nature, mechanical ventilation induces a large variety of lung responses, including oxidative stress or inflammation that can promote senescence. Moreover, mechanical stress itself can also directly activate cell senescence mechanisms. Transmission of mechanical forces from the extracellular matrix to the cell nucleus can alter the nuclear envelope, leading to changes in the mechanical properties of the nucleus and regulating gene expression [30]. Different studies have shown that abnormalities in the nuclear envelope make cells prone to enter senescence [31]. When exposed to high tidal volumes, alveolar cells change the mechanical properties of the nuclear envelope, activate the P53/P21 axis and show markers of senescence.

Overall, the complex interplay between the underlying pathology, medical interventions, and the mechanisms they trigger in critically ill patients can lead to the activation of the senescent response. This cellular response can have significant implications for the patients’ health and contribute to additional complications. Understanding these processes is essential for improving critical care and developing strategies that minimize damage by targeting these mechanisms therapeutically.

## The short- and long-term consequences of senescence

Senescence must be viewed as a homeostatic response rather than the sole consequence of aging. In fact, these pathways play key roles in tissue development and repair. Therefore, its activation during critical illness must be expected as part of the normal response to severe injuries. However, a dysregulated or persistent response may have long term, negative consequences. Moreover, the spread of SASP components can explain the systemic involvement seen in the most severe patients. Recent research has contributed to decipher the role of senescence in different syndromes commonly observed in critical care.

Senescent responses during acute lung injury have been widely characterized [32]. An increase in P53-P21 signaling has been described in experimental models of acute inflammation and in humans with ARDS or receiving mechanical ventilation [33]. Recent single-cell sequencing studies in necropsy samples show signs of DNA damage and the overexpression of the main triggers of senescence (*TP53*, *CDKN1A*) [34]. This COVID-induced senescence is seen mainly in endothelial and epithelial lung cells, although most cell types showed an increase in expression of SASP-related genes [34]. Interestingly, there is some evidence that blockade of senescence during the acute phase increases apoptosis and severity of injury [33], illustrating its early beneficial effects. It has been also proposed that the pro-inflammatory environment induced by the SASP reduces viral replication in respiratory infections [35]. However, there is also experimental and clinical evidence showing that senescence is a major player in lung fibrosis. In the context of acute lung injury, persistent activation of senescence creates a pro-fibrotic environment, and acquisition of a senescent phenotype by lung transitional or stem cells can result in impaired repair [36]. Survivors of SARS-CoV-2 infections with severe pulmonary damage show fibrotic changes that are related to telomere length [37].

A similar pattern has been observed during acute kidney injury. Renal ischemia, oxidative stress and systemic inflammation activate senescence programs, mainly in tubular epithelial cells [38]. In the acute phase, these senescent cells may promote wound repair, limit the proliferation of damaged cells and avoid a large cell loss due to apoptosis [39]. It has been shown that mutant mice lacking key senescence triggers show more severe damage in experimental models of kidney injury [40]. However, persistence of senescence results in activation of pro-fibrotic pathways that may lead to chronic kidney disease [41].

Endothelial dysfunction and injury have been described in different critical syndromes, including sepsis or acute lung, kidney or liver failure [42]. Senescent endothelial cells increase their size and number of nuclei, and release SASP-related mediators [43]. These changes promote atherosclerosis, thrombosis and vascular wall inflammation, and could explain the increased rate of cardiovascular events observed in sepsis survivors [44].

**Fig. 1:**
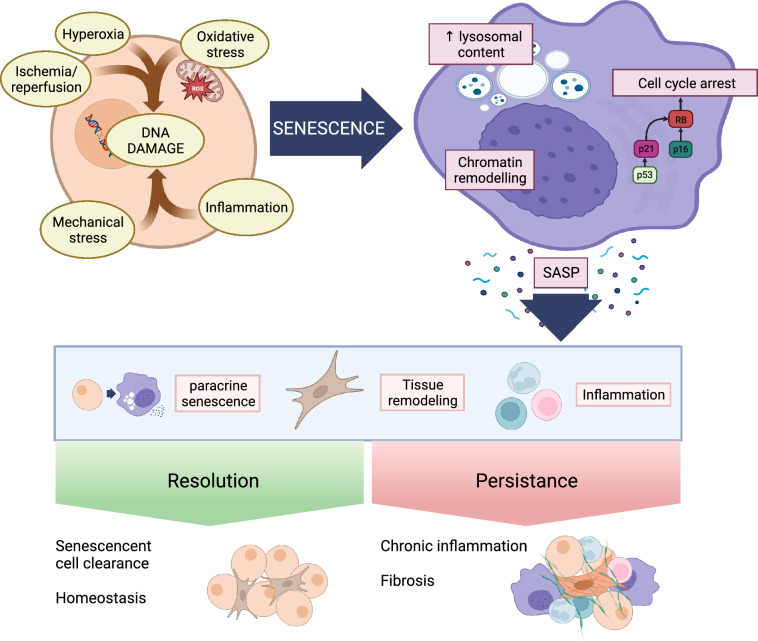
Activation of senescence and its consequences. Critically ill patients are exposed to a variety of injurious stimuli that may activate senescence mechanisms, in which P53, P21 and P16 play a key role. Senescent cells show several specific features including cell cycle arrest, changes in cell structure and release of a number of factors (known as Senescence associated secretory phenotype -SASP-) that result in systemic spread of the response and modulate inflammation and tissue remodeling. Although these mechanisms are aimed at tissue repair and clearance of damaged and senescent cells, their persistence leads to chronic inflammation and fibrosis. Created with BioRender.com

All this evidence fits into a model in which senescence is activated early as part of the acute phase response, contributing to organ homeostasis and repair. Prolonged or dysregulated senescent mechanisms can become injurious [45], favoring a prolonged pro-inflammatory and pro-fibrotic state that can spread beyond the initial site of injury (Fig. 1).

## Immunosenescence in critically ill patients

**Fig. 2:**
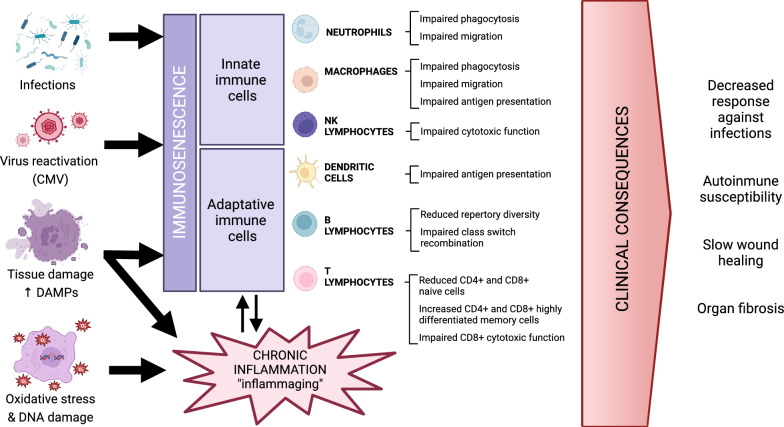
Mechanisms of immunosenescence. Triggers of senescence induce several changes in immune cell populations, favoring the shift towards a state of impaired immune response (both innate and adaptative). This “inflammaging” includes a low intensity pro-inflammatory response, in part due to the release of immune mediators from senescent cells. All these mechanisms lead to dysregulation of the immune response and establish a positive feedback loop that perpetuates this state. Created with BioRender.com

Immunosenescence is characterized by alterations of the innate and adaptative immune system producing an impairment of their normal function (Fig. 2). The main alterations involve the decrease of the repertoire diversity of naïve CD4 + and CD8 + T lymphocytes in favor to an increment of highly differentiated phenotypes. T lymphocytes with this terminally differentiated phenotype are exhausted memory lymphocytes with impaired proliferative and responsiveness capacities and a more potent proinflammatory activity. One hallmark of immunosenescence is inflammageing, described as a sterile, non-resolving, low-grade, and chronic inflammation that progressively increases with age. Inflammageing and immunosenescence processes show mutual interaction [46]. Inflammageing is caused by the increased production of pro-inflammatory cytokines mainly by innate immune cells [47, 48]. Although the underlying mechanisms of inflammageing are still unclear, one of the most studied causes is the 'Garb-aging' theory. It is based on the evidence that cellular debris accumulated over time due to cellular damage increment and progressive failure of reparation mechanisms triggers inflammation by innate immunity activation through known signaling pathways as the NF-kB transcription factor [49]. There are several circulating pro-inflammatory markers that are known to be elevated during inflammageing, contributing to SASP, as proteins associated with macrophages (sCD163, YKL-40, sCD14) and neutrophils (elastase, PR3, IL-8) and other pro-inflammatory molecules as IL-6, TNF-α and C reactive protein. Many of them have been associated to the increase of comorbidities, grater frailty and a worse outcome of some diseases [50].

Although these senescence processes were described in aging, promoted by the many contacts of the immune system with different antigens throughout life, there were also found in other situations as some chronic diseases [51, 52]. Underlying disease, ICU conditions and invasive treatments that are usually applied in critically ill patients, are known to alter the immune system homeostasis producing an immunosenescence phenotype [53, 54]. More specifically, infections, inflammation status and tissue damage associated to critical illness could be some of the specific triggers of immunosenescence.

Infections and reactivation of chronic viruses are some of the most frequent complications in critically ill patients and are known to be closely related to immunosenescence development. This response is instigated by the recognition of pathogen-associated molecular patterns (PAMPs) by the immune system, which are molecular signals derived from various pathogens. Specifically, cytomegalovirus (CMV) reactivation is the best-known exogen factor inductor of immunosenescence [55]. Up to 36% of immunocompetent patients admitted to the ICU suffer latent CMV reactivation during their stay. This is associated to an adverse outcome [56, 57]. Each viral reactivation cycle generates a subset of CMV specific T lymphocytes, with the consequent decrease of naïve T-lymphocyte repertoire and promoting a terminally differentiated phenotype and the pro-inflammatory chronic state due to the continuous immune stimulation [58, 59].

Tissue damage, also occurring due to underlying pathology and invasive treatments in critically ill patients, promotes the release of danger-associated molecular patterns (DAMPs), consisting in products from extracellular matrix and cytosol of damaged or apoptotic cells. These PAMPs/DAMPs are recognized by intracellular and extracellular toll-like receptors (TLRs) present mainly in innate immune cells generating a pro-inflammatory response. The massive release of DAMPs can also activate adaptative immune responses favoring their immunosenescence phenotype and at the same time triggering autoimmunity processes [46, 60].

Finally, immunosenescence might also be one of the mechanisms driving the accumulation of senescent cells in tissues both during critical illness and aging. These changes in immune cell function might render them unable to reach the local senescent cells and clear the tissue from them, which will further exacerbate the cycle of systemic inflammation [61, 62]. All these described processes that frequently occur in critically ill patients, would act as a positive feedback loop, perpetuating the systemic inflammation which leads to a worse prognosis of these patients.

## Interaction between senescence and aging

Senescence is part of physiological aging, but, as previously discussed, can also be triggered by other stimuli. This implies that, although correlated, senescence and aging can be independent phenomena. The crosstalk between senescence and aging is only partially known.

Activation of senescence molecular mechanisms and accumulation of senescent cells increase with age. Repeated exposure to injurious stimuli and telomer shortening due to cell replication are responsible for this phenomenon along the lifespan. One of the consequences of this activation is the persistence of a low intensity proinflammatory response due to the concurrent SASP [63]. In old critically ill patients, this increased proinflammatory milieu can impair tissue repair and recovery, thus contributing to the poor outcome in this population [64]. In addition, this increased activation of senescence may modify the acute response to new injuries. Aged animals show an attenuated inflammatory response in experimental models of sepsis, with dysfunctional cell populations and only minor changes in the expression of proinflammatory genes [65]. It is unclear how these two counteracting conditions (activation of senescence in baseline conditions and decreased senescent response after a new stimulus) modulate the pathogenesis of critical diseases.

## Monitoring senescence in critically ill patients

Given the dual nature, adaptative and pathogenetic, of the senescence response to acute injuries, monitoring becomes a critical issue to identify therapeutic time windows in which pharmacological manipulation achieves the maximal benefit. This task is difficult due to the lack of specific and universal markers. Moreover, all the approaches based on monitoring of SASP components [66] are invalid in critically ill patients, due to the massive release of proinflammatory mediators triggered by the underlying disease.

A more specific approach to systemic monitoring of senescence could be achieved using multiparametric techniques. Sequencing RNA from circulating cells allows the quantification of several proposed transcriptomic signatures in peripheral blood [67, 68]. By quantifying the expression of several genes, these signatures can be synthetized in a single value. Recently, a detailed proteomic analysis has been able to quantify aging (not senescence) at an organ-specific level by measuring tissue specific protein signatures [69]. Finally, flow cytometry allows the identification and quantification of senescence markers at a cell level [70]. However, the validity of these approaches must be confirmed in a complex scenario such as critical care.

Monitoring senescence could help to define optimal windows of opportunity to treat patients with drugs that modify the senescent response (see below). One could speculate that this kind of drugs should be tested in enriched clinical trials, in which only those patients with a clear senescent phenotype should be included and treated. This kind of precision medicine, that stems from the underlying pathogenetic mechanisms and applies phenotype-specific therapies, constitutes the future of critical care [71].

## Therapeutic modulation of senescence: senotherapeutics

Senescent cells depend on the activation of pro-survival and anti-apoptotic pathways to avoid cell death. Therefore, any drug that inhibits these biochemical routes will trigger the selective apoptosis of senescent cells. Most of these so-called senolytics target intracellular factors involved in regulation of apoptosis. For instance, BCL-2 inhibitors, such as navitoclax or venetoclax, have been widely used in experimental models to remove senescent cells. This approach has shown beneficial results in models of lung fibrosis [72]. Recently, it has been shown that navitoclax decreases viral load, systemic inflammation and SASP markers in a model of COVID-19 in aged hamsters, in line with its senolytic activity [73]. Other pathways can be blocked using kinase inhibitors. Among these, dasatinib, a non-selective inhibitor of tyrosin-kinases and SRC-kinases, has been used in experimental models of sepsis and acute lung injury with favorable results [74, 75]. However, these drugs have significant toxicities, including peripheral blood cytopenias and cardiovascular events that raise concerns on their use in critically-ill patients. As an alternative, flavonoids may have senolytic properties, at least in part due to their effect as BCL2-inhibitors, and have a better safety profile. Quercetin and fisetin have been used in models of senescence, alone or combined with other drugs. There are several reports on the use of flavonoids in critical care [76, 77]. Targeting HSP-90, a pro-survival protein involved in sepsis and acute lung injury, also promotes apoptosis by facilitating the elimination of the pro-survival kinase AKT. Several drugs, such as geldanamycin, ansamycin or resorcinol act at this level, and have shown benefits in experimental models of acute lung injury or sepsis [78, 79].

Other widely used drugs have been repurposed as senolytics. Digoxin and other cardiac glucosides promote the death of senescent cells [80, 81], as these cannot cope with the changes in intracellular concentrations of pH and calcium triggered by the drug. This drug decreases lung fibrosis after bleomycin administration by clearance of senescent cells [81]. However, no clinical data are available. It must be taken into account that these findings are limited by the pleiotropic nature of the tested inhibitors and their targets, thus precluding any firm conclusion regarding their specific effects on senescence. In addition, there is no evidence of benefits in critical care settings.

A different approach is the use of drugs to inhibit SASP. These drugs are termed senomorphics. Most of the drugs that block the inflammatory response, such as rapamycin or NF-kB or JAK-STAT inhibitors, may fall in this category. In critically ill patients, where a pro-inflammatory response is usually part of the pathogenesis of the disease, these drugs may have potential benefits. However, as in the case of senolytics, it is not clear that these benefits are linked to a specific effect on senescence.

Again, several drugs have senomorphic effects. Metformin, at least in part by their effects as blocker of the NF-kB and Nrf2 pathways, decreases experimental lung injury caused by endotoxin or alveolar overdistension [82], and the development of fibrosis after bleomycin-induced inflammation [83]. Clinical observational data has shown a reduction of mortality in patients with sepsis and diabetes [84] or pneumonia, but, again, the link with senescence has not been tested. Similarly, statins can modulate SASP by upregulating several sirtuins, a family of proteins that inhibit senescence. Several observational studies and trials have addressed the use of statins in critically ill patients. Although some works report benefits in survival and long-term sequels [85–87], randomized trials have not supported their use in unselected populations in critically ill patients [88–90].

Given the previously proposed model of senescence in critical illness, timing of these treatments is essential. Early blockade of senescence may promote harm, as it blocks a homeostatic mechanism. Most of the potential benefits of senescence-targeting drugs are related the avoidance of late sequels. Preclinical research has also pointed to these benefits, usually in experimental models of chronic diseases such as lung or kidney secondary fibrosis [38]. This highlights the need for animal models of late sequels of critical illness to test the proposed therapeutic approaches. In these experimental models, time windows can be defined a priori. However, clear identification of the transition from early to late phases of the disease in patients may be difficult, and would probably imply the use of systemic or local biomarkers, as previously proposed.

## Conclusions

Despite the strong link between aging and senescence, the latter is now understood as a cell mechanism activated in response to a variety of stimuli. Aging implies the continued activation of the senescence machinery, but in critically ill patients, senescence may be activated irrespective of patients’ age to play a key role in tissue homeostasis by increasing cell resilience to injury, decreasing apoptosis and promoting tissue remodeling and clearance of injured cells. As virtually all the homeostatic mechanisms during critical illness, the continued, dysregulated activation of senescence favors tissue damage, usually caused by persistent inflammation and fibrosis. This raises the hypothesis that the so-called Post-intensive care syndrome can be, at least in part, the manifestation of accelerated aging [45]. Knowledge of these senescence pathways can allow their monitoring and pharmacological manipulation, probably in specific time windows, to improve the outcome of critical care patients.

## Data Availability

Not applicable.

## Abbreviations

CMV: Cytomegalovirus
DAMPs: Danger-associated molecular patterns
DDR: DNA damage response
PAMPs: Pathogen-associated molecular patterns
ROS: Reactive oxygen species
SASP: Senescence-associated secretory phenotype
TLRs: Toll-like receptors
